# REDD1-dependent GSK3β signaling in podocytes promotes canonical NF-κB activation in diabetic nephropathy

**DOI:** 10.1016/j.jbc.2025.108244

**Published:** 2025-01-27

**Authors:** Siddharth Sunilkumar, Esma I. Yerlikaya, Ashley VanCleave, Sandeep M. Subrahmanian, Allyson L. Toro, Scot R. Kimball, Michael D. Dennis

**Affiliations:** Department of Cellular and Molecular Physiology, Penn State College of Medicine, Hershey, Pennsylvania, USA

**Keywords:** diabetic nephropathy, inflammation, podocyte, DDIT4, glycogen synthase kinase 3 (GSK-3)

## Abstract

Increasing evidence supports the role of an augmented immune response in the early development and progression of renal complications caused by diabetes. We recently demonstrated that podocyte-specific expression of stress response protein regulated in development and DNA damage response 1 (REDD1) contributes to activation of the pro-inflammatory transcription factor NF-**κ**B in the kidney of diabetic mice. The studies here were designed to define the specific signaling events whereby REDD1 promotes NF-**κ**B activation in the context of diabetic nephropathy. Streptozotocin (STZ)-induced diabetes promoted activation of glycogen synthase kinase 3**β** (GSK3**β**) in the kidney, which was prevented by REDD1 ablation. REDD1 was necessary and sufficient to enhance GSK3**β** activity in human podocyte cultures exposed to hyperglycemic conditions. GSK3**β** suppression prevented NF-**κ**B activation and normalized the expression of pro-inflammatory factors in podocytes exposed to hyperglycemic conditions. In the kidneys of diabetic mice and podocytes exposed to hyperglycemic conditions, REDD1-dependent GSK3**β** signaling promoted activation of the inhibitor of **κ**B (IκB) kinase (IKK) complex upstream of NF-**κ**B. GSK3**β** knockdown in podocytes exposed to hyperglycemic conditions reduced macrophage chemotaxis. Similarly, in diabetic mice treated with a GSK3 inhibitor, immune cell infiltration in the kidneys was reduced. Overall, the data support a model wherein hyperglycemia amplifies the activation of GSK3**β** in a REDD1-dependent manner, leading to canonical NF-**κ**B signaling and an augmented renal immune response in diabetic nephropathy.

Diabetic nephropathy (DN) is a major microvascular complication associated with increased mortality in diabetic patients, as it is the leading cause of end-stage renal disease (1). Despite recent advancements in therapeutic approaches to achieve improved blood glucose control, the health burden of DN remains significant (2). Presently, an improved understanding of the precise signaling events whereby diabetes and hyperglycemic conditions cause DN pathophysiology is needed to facilitate the development of improved therapeutics that more specifically address the cause of kidney disease. While the pathophysiology of diabetes is complex, it is well-accepted that both chronic low-grade inflammation and immune system activation are key drivers of DN pathogenesis.

In sterile inflammation, activation of the nuclear factor kappa B (NF-κB) family of transcription factors promotes the expression of inflammatory cytokines and chemokines that recruit immune cells into the kidney (3). NF-κB stimulates the expression of pro-inflammatory factors and chemokines, including interleukin 1β (IL-1β) and C-C motif ligand 2 (CCL2/MCP-1). Extensive investigation supports a role for NF-κB activation and increased cytokine production in the pathogenesis of DN. Indeed, unbiased RNA expression profiles from human kidney biopsies support the activation of specific NF-κB promoter regions in the inflammatory response of progressive DN (4). Furthermore, drug therapies and natural products have been shown to exert protective effects in the development and progression of DN by attenuating NF-κB activation (5). NF-κB activity is controlled by an inhibitor of κB (IκB), which sequesters the transcription factor in the cytoplasm. IκB kinase (IKK) phosphorylates IκB to promote its proteolysis, IKK-dependent phosphorylation of NF-κB at S536, and NF-κB nuclear translocation (6). IKK is composed of two kinase subunits (IKKα/β) and the regulatory subunit NEMO. While much is known about canonical and non-canonical NF-κB signaling in immune cells, the specific signaling events through which diabetes activates this pathway in renal cells require further elucidation.

The serine-threonine protein kinase glycogen synthase kinase 3 (GSK3) regulates various cellular pathways, including glucose metabolism, cell proliferation and death, oxidative stress, and inflammation (7, 8, 9, 10). GSK3 exists as two isoforms (GSK3α and GSK3β), which serve different biological purposes despite having similar structures. Within the kidney, glomerular podocytes exhibit dominant expression of GSK3β *versus* GSK3α (9, 11, 12). Evidence supports podocyte-specific GSK3 signaling as a crucial regulator of kidney function (13). In glomerular podocytes of embryonic or adult mice, deletion of both GSK3α and GSK3β results in severe podocyte damage, glomerulosclerosis, and significant proteinuria. In the context of DN, elevated GSK3β expression has been observed within glomeruli of patients with diabetes (12) and increased expression of GSK3β in urinary exfoliated renal cells from diabetic patients is a predictive biomarker of disease progression (7). Increased expression of GSK3β has also been observed within podocytes in preclinical rodent models of diabetes (7, 9, 12, 14) and pharmacologic inhibition of GSK3β has shown promise in ameliorating diabetic kidney injury (9, 14, 15, 16, 17, 18, 19). While its role in regulating diabetes-induced oxidative stress injury is well studied (12, 14), the role of GSK3β in regulating inflammation in the context of DN remains unclear.

The stress response protein REDD1 (Regulated in Development and DNA Damage 1; also known as DDIT4 or RTP801) has emerged as a promising therapeutic target to address the development of diabetic complications (20, 21). In the context of DN, independent investigations have shown that REDD1 protein abundance is increased in the kidneys of patients with diabetes and preclinical animal models of diabetes (14, 22, 23). Our laboratory recently demonstrated that germline (14) or podocyte-specific deletion (23) of REDD1 attenuates kidney injury and filtration function defects in diabetic mice. While REDD1 is best known as a dominant regulator of the protein kinase complex mTORC1 (mammalian target of rapamycin in complex 1) (19), REDD1 also promotes GSK3β activation by suppressing its inhibitory phosphorylation. In the past decade, investigations by us and others support a key role for REDD1 in mediating NF-κB activation and inflammation in disease pathology (20, 21). However, the role of REDD1 in diabetes-induced renal inflammation and immune response activation has not been fully elucidated. Herein, we investigated the role of REDD1-dependent GSK3β signaling in the activation of canonical NF-κB signaling in renal inflammation caused by diabetes.

## Results

### REDD1 was required for diabetes-induced GSK3**β** activation in the kidney

**Figure 1:**
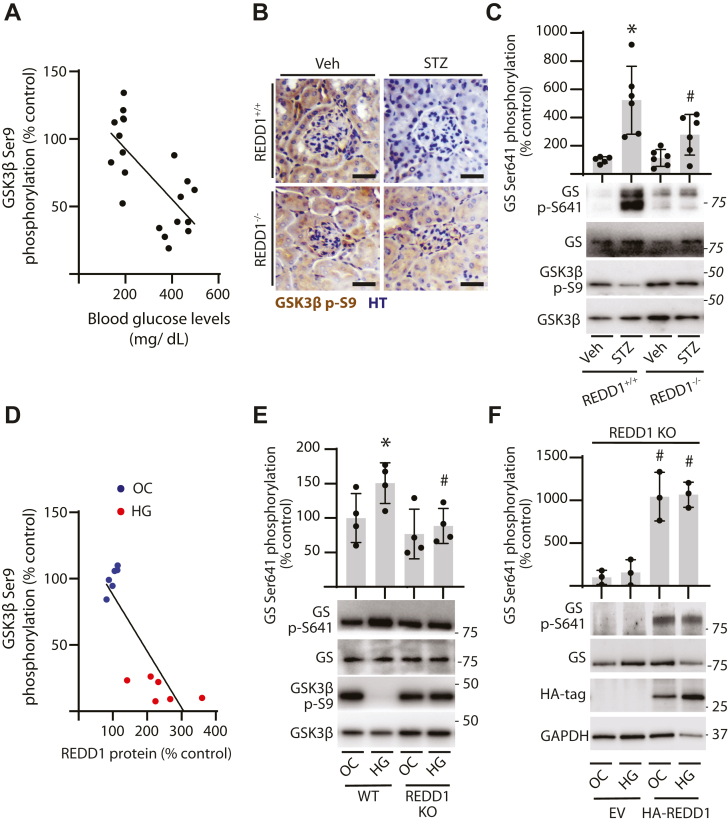
**REDD1 promoted GSK3β activation in the kidneys of diabetic mice.***A–C*, REDD1^+/+^ and REDD1^−/−^ mice were administered streptozotocin (STZ) to induce diabetes. Nondiabetic control mice were administered vehicle (Veh). Protein analysis was performed after 16 weeks of diabetes. The correlation between fasting blood glucose concentration and renal GSK3β phosphorylation at S9, as determined by Western blotting (14), was estimated (Pearson r = −0.694). *A*, phosphorylation of GSK3β at S9 (*brown*) was examined in renal sections by immunohistochemistry. *B*, representative micrographs (scale bar 25 μm) are shown with nuclei counterstained by hematoxylin (HT, *blue*). Phosphorylation of glycogen synthase (GS) at S641 and GSK3β at S9 was determined in renal cortical tissue homogenates by Western blotting. *C*, representative blots are shown with protein molecular mass in kDa indicated at the *right* of each blot. *D–F*, Wild-type (WT), and REDD1 knockout (KO) human CIHP-1 cells were exposed to culture media containing either 30 mM glucose (HG) or 5 mM glucose plus 25 mM mannitol as an osmotic control (OC) for 48 h. Correlation between phosphorylation of GSK3β at S9 and REDD1 protein abundance (*D*), as determined by Western blotting (14), was estimated (Pearson r = −0.841). GS phosphorylation at S641 and GSK3β phosphorylation at S9 were determined in WT and REDD1 KO cell lysates by Western blotting (*E*). Phosphorylation of GS at S641 was evaluated in REDD1 KO cell lysates expressing either an empty vector control (EV) or hemagglutinin (HA)-tagged REDD1 (*F*). Individual data points are plotted and presented as means ± SD. Differences between groups were identified by two-way ANOVA. ∗*p* < 0.05 *versus* Veh or OC; #*p* < 0.05 *versus* REDD1^+/+^, WT, or EV. Spearman correlation analysis was performed in *A* and *D*.

GSK3β activity is increased in the kidneys of diabetic patients and preclinical rodent models of diabetes (7, 14, 17, 18). In support of these observations, the inhibitory phosphorylation of GSK3β at S9 in kidney cortical homogenates was negatively correlated with increased fasting blood glucose levels in diabetic mice (Fig. 1*A*; Pearson r = −0.694) (14). We previously demonstrated that REDD1-dependent GSK3β dephosphorylation at S9 was associated with podocytopenia in diabetic mice (14). In the kidney of diabetic REDD1^+/+^ mice, GSK3β S9 phosphorylation was reduced (Fig. 1, *B* and *C*) coincident with increased phosphorylation of the GSK3β substrate glycogen synthase (GS) at S641 (Fig. 1*C*). Diabetes-induced changes in phosphorylation of GSK3β and GS in the kidney were attenuated in REDD1^−/−^ mice. Similarly, in CIHP-1 podocyte cultures exposed to hyperglycemic conditions, increased REDD1 protein abundance was negatively correlated with GSK3β phosphorylation at S9 (Fig. 1*D*; Pearson r = − 0.841) (14). REDD1 expression in podocytes was required for enhanced phosphorylation of GS upon exposure to hyperglycemic conditions (Fig. 1*E*). Moreover, HA-REDD1 rescue enhanced GS phosphorylation in REDD1-deficient cells (Fig. 1*F*). The data support that REDD1 acts to activate GSK3β in the kidneys of diabetic mice and in podocytes exposed to hyperglycemic conditions.

### REDD1-dependent GSK3**β** signaling promoted NF-**κ**B activation in podocytes

**Figure 2:**
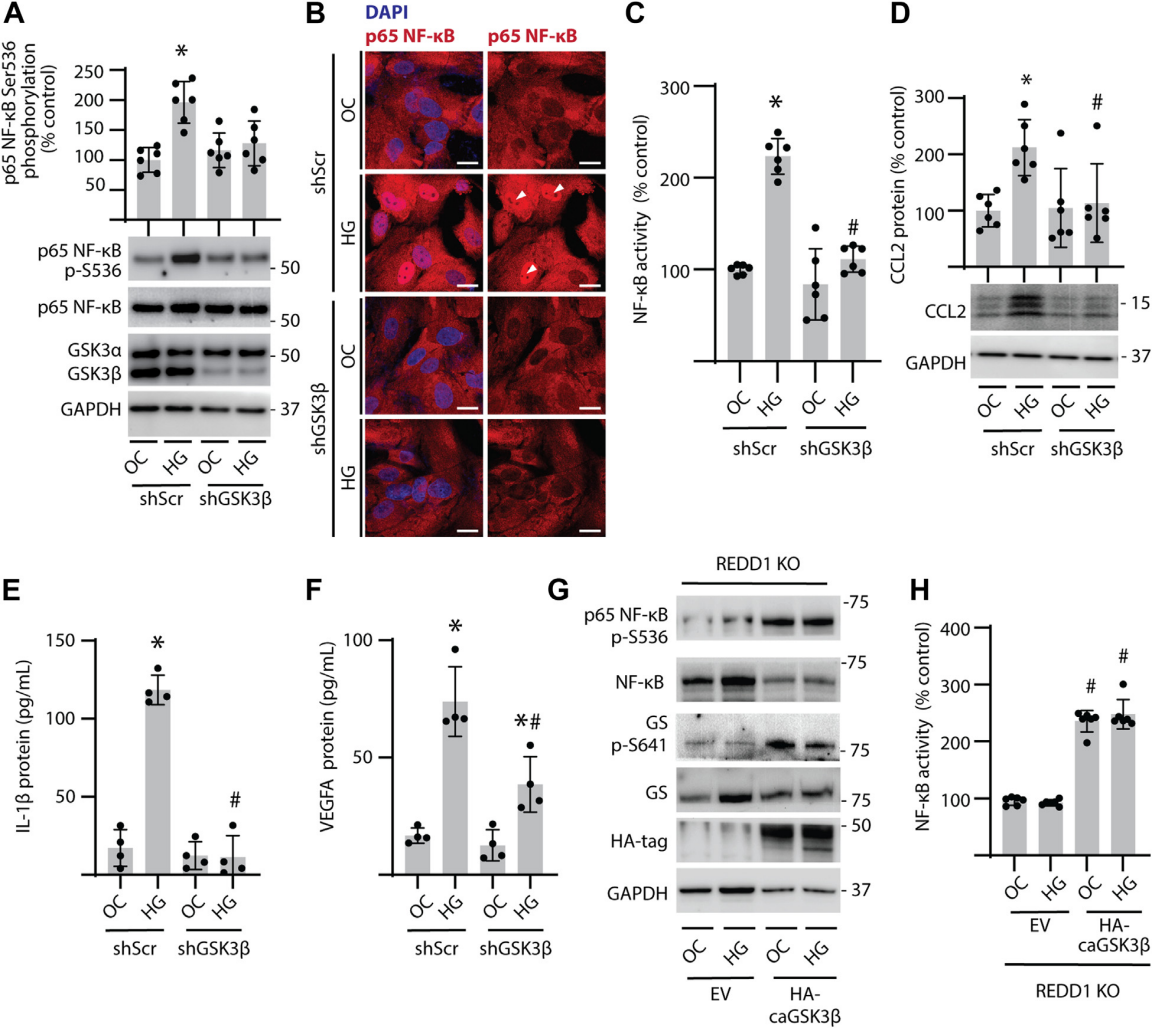
**GSK3β promoted NF-κB activation in podocytes exposed to hyperglycemic conditions.***A–F*, GSK3β was knocked down in human CIHP-1 cells by stable expression of a shRNA (shGSK3β). Control cells expressed a scramble shRNA (shScr). Cells were exposed to culture media containing either 30 mM glucose (HG) or 5 mM glucose plus 25 mM mannitol as an osmotic control (OC) for 48 h. NF-κB phosphorylation at S536 and GSK3α/β protein abundance was determined by Western blotting (*A*). Representative western blots are shown with protein molecular mass indicated at the *right* of the blot. Nuclear localization of NF-κB p65 (*white arrowheads*) was evaluated by immunofluorescence (scale bar 25 μm) (*B*). Nuclei were counterstained with DAPI (*blue*). NF-κB reporter activity was measured in cell lysates by dual luciferase assay (*C*). CCL2 protein abundance was determined in cell lysates by western blotting (*D*). Protein levels of IL-1β (*E*) and VEGFA (*F*) were determined in cell culture supernatants using ELISA. *G* and *H*, REDD1 knockout (KO) CIHP-1 cells expressing either an empty vector (EV) control or an HA-tagged constitutively active GSK3β S9A (HA-caGSK3β) were exposed to OC or HG for 48 h. HA-GSK3β and phosphorylation of NF-κB and GS were evaluated by Western blotting (*G*). NF-κB luciferase reporter activity was measured in cell lysates (*H*). Individual data points are plotted and presented as means ± SD. Differences between groups were identified by two-way ANOVA with pairwise comparisons made using Tukey's test for multiple comparisons. ∗*p* < 0.05 *versus* OC; #*p* < 0.05 *versus* shScr or EV.

To investigate the influence of GSK3β on NF-κB activation, GSK3β-deficient podocytes were generated by stable shRNA expression (Figs. 2*A* and S1, *A*–*C*). In podocytes exposed to hyperglycemic conditions, NF-κB phosphorylation at S536 (Figs. 2*A* and S1*D*), nuclear localization (Fig. 2*B*), and activity (Fig. 2*C*) was increased as compared to an osmotic control. GSK3β knockdown prevented an increase in the phosphorylation and nuclear localization of NF-κB in response to hyperglycemic conditions, and normalized activity of the transcription factor. Similarly, hyperglycemic conditions increased CCL2 protein abundance in podocytes, which was prevented by GSK3β knockdown (Fig. 2*D*). Hyperglycemic conditions increased IL-1β (Fig. 2*E*) and VEGFA (Fig. 2*F*) protein in cell culture supernatants from wild-type podocytes, which was prevented by GSK3β knockdown. To define the role of GSK3β in REDD1-dependent NF-κB activation, a constitutively active GSK3β S9A variant (caGSK3β) was expressed in REDD1-deficient podocytes. Expression of caGSK3β enhanced phosphorylation of both NF-κB and GS and was sufficient to promote NF-κB activity in REDD1-deficient podocytes, independently of hyperglycemic conditions (Fig. 2, *G* and *H*). Together, the data support a role for REDD1-mediated GSK3β signaling in podocyte NF-κB activation and proinflammatory cytokine/chemokine expression in response to hyperglycemic conditions.

### GSK3**β** was required for canonical NF-**κ**B signaling in response to hyperglycemic conditions

**Figure 3:**
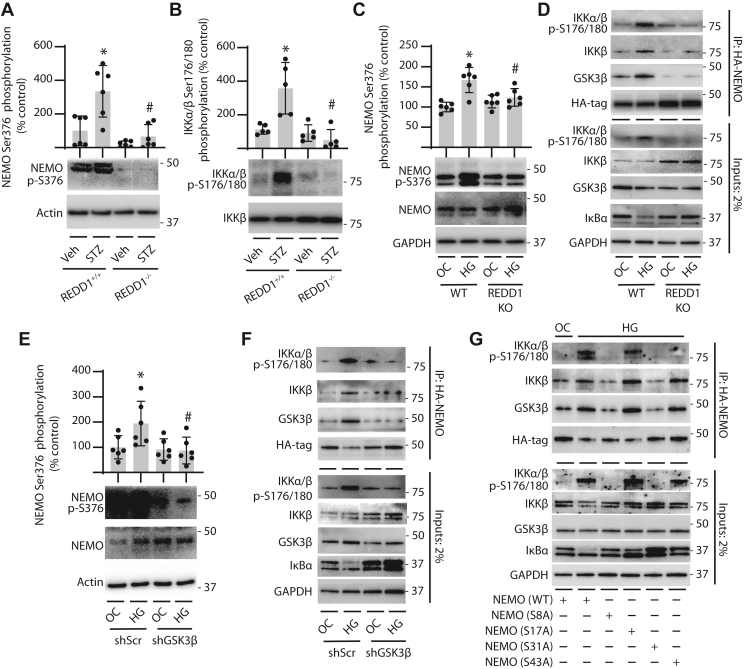
**REDD1 and GSK3β were required for IKK activation.***A* and *B*, REDD1^+/+^ and REDD1^−/−^ mice were administered either streptozotocin (STZ) to induce diabetes or a vehicle (Veh) control. Phosphorylation of NEMO at S376 (*A*) and IKKα/β at S176/180 (*B*) was determined by western blotting. Representative blots are shown. Molecular mass in kDa is indicated at *right* of each blot. *C* and *D*, wild-type (WT) and REDD1 knockout (KO) CIHP-1 cells were exposed to media containing either 30 mM glucose (HG) or 5 mM glucose plus 25 mM mannitol as an osmotic control (OC) for 48 h. NEMO phosphorylation was evaluated in cell lysates by western blotting (*C*). HA-tagged NEMO (HA-NEMO) was expressed in WT and REDD1 KO cells (*D*). HA-NEMO was immunoprecipitated from cell lysates by affinity purification. Cell lysate (2% of input) and the HA-tag immunoprecipitate (IP) were examined by western blotting. *E* and *F*, CIHP-1 cells expressing either a shRNA targeting GSK3β (shGSK3β) or a control shRNA (shScr) were exposed to HG or OC for 48 h. NEMO phosphorylation was evaluated in cell lysates (*E*). HA-NEMO was expressed in CIHP-1 cells and immunoprecipitation was performed (*F*). *G*, CIHP-1 cells were transfected to express HA-tagged NEMO or NEMO S8A, S17A, S31A, or S43A variants as indicated. Data are presented as means ± SD. Differences were identified by two-way ANOVA, and pairwise comparisons were made using Tukey's test for multiple comparisons. ∗*p* < 0.05 *versus* Veh or NG; #*p* < 0.05 *versus* REDD1^+/+^, WT or shScr.

In the kidney of REDD1^+/+^ mice, diabetes enhanced NEMO phosphorylation at S376 relative to actin (Fig. 3*A*) and IKKα/β autophosphorylation at S176/180 (Fig. 3*B*). NEMO and IKKα/β phosphorylation were similar in the kidneys of non-diabetic REDD1^+/+^ and REDD1^−/−^ mice. Diabetes-induced changes in NEMO and IKKα/β phosphorylation were not seen in REDD1^−/−^ mice. REDD1 was also required for enhanced NEMO phosphorylation in podocytes exposed to hyperglycemic conditions (Fig. 3*C*). Hyperglycemic conditions promoted NEMO co-immunoprecipitation (IP) with IKKβ, supporting IKK complex assembly (Fig. 3*D*). In addition to promoting IKKβ binding to NEMO, GSK3β co-IP with NEMO was also enhanced in response to hyperglycemic conditions in coordination with reduced IκBα protein abundance. Notably, REDD1 was required for increased binding of both IKKβ and GSK3β with NEMO, and the reduction in IκBα protein.

Consistent with a role for REDD1 in IKK activation, GSK3β knockdown also prevented NEMO phosphorylation, IKK complex assembly, and the reduction in IκBα in podocytes exposed to hyperglycemic conditions (Figs. 3, *E* and *F* and S1*D*). A previous report identified GSK3β phosphorylation sites within the N-terminal domain of NEMO that were essential for NF-κB activation (3). Whereas hyperglycemic onditions promoted IKKβ co-IP with wild-type NEMO, a similar effect was not observed with NEMO S8A or NEMO S31A variants (Fig. 3*G*). Additionally, IκBα protein abundance was reduced in cells expressing wild-type NEMO upon exposure to hyperglycemic conditions; however, IκBα levels were not reduced in response to hyperglycemic conditions in cells expressing NEMO S8A, S17A, S31A, or S43A. Together the data support that activation of REDD1-dependent GSK3β signaling is required for IKK complex assembly and activity in response to hyperglycemic conditions.

### GSK3 inhibition attenuated NF-**κ**B activation in the kidney of diabetic mice

**Figure 4:**
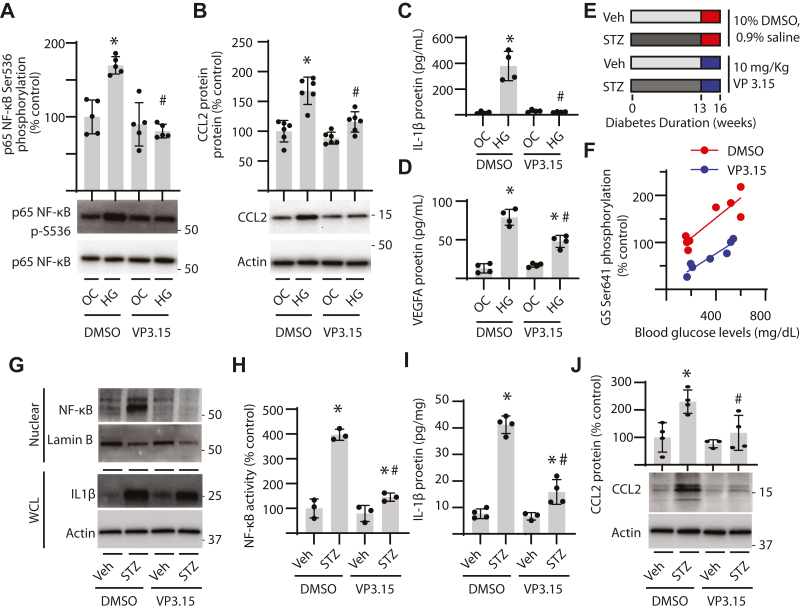
**GSK3 inhibition suppressed renal inflammation in diabetic mice.***A–D*, CIHP-1 cells were exposed to media containing 30 mM glucose (HG) or an osmotic control (OC) containing 5 mM glucose plus 25 mM mannitol in the presence of the GSK3 inhibitor VP3.15 (1 μM) or a vehicle control (0.1% DMSO). NF-κB phosphorylation at S536 (*A*) and CCL2 protein abundance (*B*) was determined in cell lysates by western blotting. Representative western blots are shown with protein molecular mass indicated at the *right* of the blot. Protein levels of IL-1β (*C*) and VEGFA (*D*) released into cell culture supernatants were determined using ELISA. *E–J*, mice were administered streptozotocin (STZ) to induce diabetes or a vehicle control (Veh). Kidney tissue was analyzed after 16 weeks of diabetes. GSK3 inhibition was carried out during the last 3 weeks by administering VP3.15 (10 mg/kg in 10% DMSO, 0.9% NaCl) or a vehicle control (DMSO) daily. *F*, correlation between blood glucose levels and GS phosphorylation at S641 was estimated. Nuclear NF-κB abundance was determined in renal tissue homogenates by Western blotting (*G*). Lamin B was used as a control for nuclear isolation. NF-κB activity in nuclear isolates was assayed by DNA-binding ELISA (*H*). IL-1β protein abundance in renal homogenates was visualized by Western blotting (*G*) and was quantified by ELISA (*I*). CCL2 protein abundance in kidney tissue homogenates was evaluated by Western blotting (*J*). Individual data points are plotted and presented as means ± SD. Differences between groups were identified by two-way ANOVA with pairwise comparisons made using Tukey's test for multiple comparisons. ∗*p* < 0.05 *versus* OC or Veh; #*p* < 0.05 *versus* shScr or DMSO. Spearman correlation analysis was carried out in *F*.

Pharmacological inhibition of GSK3 with VP3.15 prevented increased phosphorylation of GS and NF-κB in podocytes exposed to hyperglycemic conditions (Fig. 4*A*). Increased CCL2 protein abundance in cells exposed to hyperglycemic conditions was also attenuated by GSK3β inhibition (Fig. 4*B*). Co-treatment with VP3.15 attenuated secretion of IL-1β (Fig. 4*C*) and VEGFA (Fig. 4*D*) in podocytes exposed to hyperglycemic conditions. To investigate the role for GSK3β in diabetic kidney disease, mice were treated with VP3.15 (Fig. 4*E*). GS phosphorylation in the kidneys was enhanced in coordination with increased blood glucose concentrations in diabetic mice (Fig. 4*F*) (14). NF-κB nuclear localization (Fig. 4*G*) and activity (Fig. 4*H*) were increased in the kidneys of diabetic mice. VP3.15 attenuated GS phosphorylation and reduced NF-κB nuclear localization and activity in the kidneys of diabetic mice. Diabetes also increased IL-1β (Fig. 4, *G* and *I*) and CCL2 (Fig. 4*J*) in the kidneys, which was suppressed by VP3.15 treatment.

### Diabetes-induced immune cell infiltration of the kidney was prevented by GSK3 inhibition

**Figure 5:**
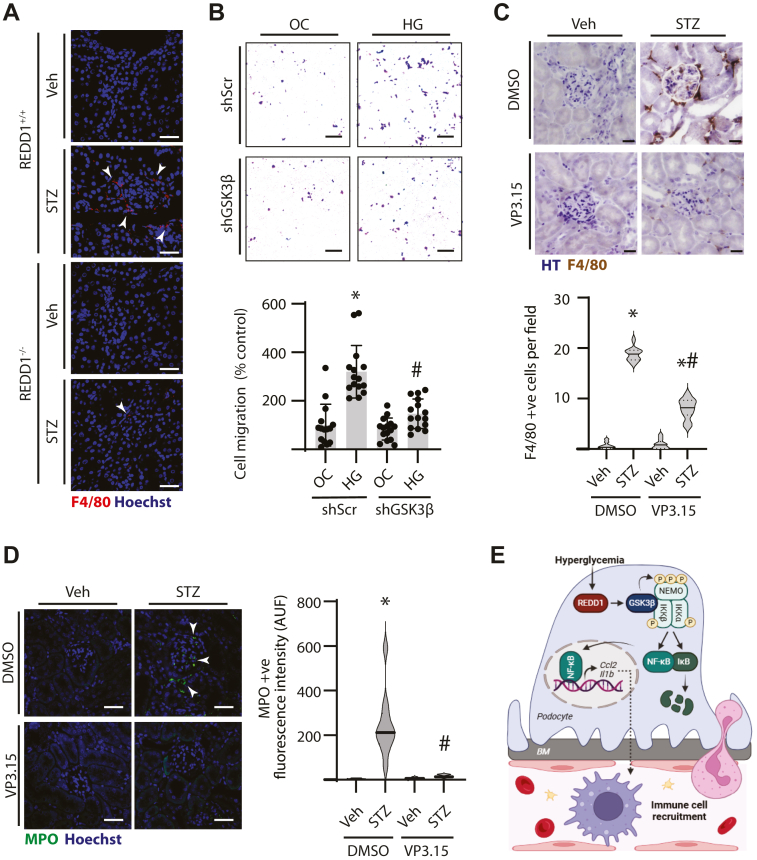
**GSK3β inhibition attenuated diabetes-induced renal immune cell infiltration.***A*, renal tissue sections from diabetic (STZ) or non-diabetic (Veh) REDD1^+/+^ and REDD1^−/−^ mice were immunolabelled for F4/80 positive cell infiltrates (*red*) and counterstained with Hoechst33342 (*blue*). *B,* CIHP-1 cells that expressed either a shRNA targeting GSK3β (shGSK3β) or a scramble shRNA control (shScr) were exposed to media containing 30 mM glucose (HG) or an osmotic control (OC) containing 5 mM glucose plus 25 mM mannitol. Transwell migration assay was used to evaluate chemotaxis in a co-culture model with CIHP-1 and THP-1 macrophages. Macrophages were stained with crystal violet and cells that migrated across the transwell were counted. *C*, mice were administered streptozotocin (STZ) or vehicle control (Veh) and administered VP3.15 as depicted in Figure 4*E*. Renal cortical sections were immunolabeled for F4/80 (*brown*) and nuclei were counterstained with hematoxylin (HT, *blue*). F4/80+ cells were quantified (15 fields per kidney, n = 3). Tissue sections were immunolabeled for myeloperoxidase (MPO; *green*) and counterstained with DAPI (*blue*). The fluorescence intensity of MPO + staining was quantified (5 fields per kidney, n = 3). Representative micrographs are shown (scale bar 50 μm). Individual data points are plotted, and differences are identified by two-way ANOVA with pairwise comparisons made using Tukey's test for multiple comparisons. ∗*p* < 0.05 *versus* OC or Veh; #*p* < 0.05 *versus* shScr or DMSO. *E*, working model illustrates the role of REDD1-dependent GSK3 signaling in the development of renal inflammation in diabetic nephropathy. Hyperglycemia enhances REDD1-dependent GSK3β signaling in podocytes, resulting in activation of the canonical NF-κB pathway, increased cytokine and chemokine production, and kidney immune cell infiltration. BM, basement membrane; P, phosphorylation.

Prior literature supports an immunomodulatory role for podocytes in the kidney (24, 25, 26). REDD1 deletion prevented F4/80+ immune cell infiltration in the kidneys of diabetic mice (Fig. 5*A*). To evaluate macrophage chemotaxis by podocytes, a transwell migration assay was performed. Macrophage transmigration was increased when co-cultured with podocytes exposed to hyperglycemic conditions *versus* an osmotic control (Fig. 5*B*). GSK3β knockdown in podocytes exposed to hyperglycemic conditions reduced macrophage chemotaxis. Consistent with the cell culture transwell migration assay, pharmacological inhibition of GSK3β by VP3.15 also reduced a diabetes-induced increase in F4/80 +ve (Fig. 5*C*) and myeloperoxidase (MPO) +ve (Fig. 5*D*) immune cell infiltration into glomeruli of diabetic mice.

## Discussion

Studies from the last 2 decades support the critical role of inflammation in the etiology of DN. Independent investigations including studies by our group have demonstrated a key role for the stress response protein REDD1 in promoting inflammation (20, 21). Herein, we investigated the signal transduction mechanisms downstream of REDD1 that contribute to renal inflammation in the context of diabetes. REDD1 and GSK3β were both necessary for IKK complex activation, NF-κB nuclear localization, and the inflammatory response in the kidneys of diabetic mice. Overall, the studies support a model wherein REDD1-dependent GSK3β signaling in podocytes promotes canonical NF-κB activation and the production of pro-inflammatory factors that drive renal immune cell recruitment in DN (Fig. 5*E*).

GSK3β activation in urinary exfoliated cells predicts DN progression (7). Indeed, reduced inhibitory phosphorylation of GSK3β at S9 and activation of its kinase activity has been reported in multiple preclinical models of renal injury, including diabetes (27). REDD1 acts to suppress the inhibitory phosphorylation of GSK3β, resulting in enhanced activity of the kinase (28). More precisely, REDD1 recruits protein phosphatase 2A to dephosphorylate Akt at T308, which attenuates Akt-dependent phosphorylation of several substrates, including GSK3β. GSK3β phosphorylation at S9 reduces its kinase activity by obstructing substrate recognition (29). Data presented here extends from prior studies (14, 30) demonstrating that REDD1 is required for suppression of GSK3β phosphorylation and the subsequent increase in GSK3 kinase activity in both the kidneys of diabetic mice and in podocytes exposed to hyperglycemic conditions. We previously demonstrated that diabetes-induced REDD1 promotes renal oxidative stress and podocyte loss by suppressing the Nrf2 antioxidant response in a GSK3β-dependent manner (14). Thus, REDD1-dependent GSK3β activation contributes to both diabetes-induced oxidative stress and inflammation in the kidney.

GSK3β-targeted therapeutics have protective effects on urinary albumin excretion in models of both type 1 and type 2 diabetes (14, 17, 18). Microdose administration of the well-known GSK3 inhibitor lithium protects against renal impairment in diabetic mice (18), as well as in models of folic acid-induced nephropathy (31) by enhancing the Nrf2-mediated antioxidant response. Consistent with the studies herein, GSK3β inhibition attenuates inflammation in preclinical disease models, including models of Alzheimer’s disease, psoriasis, and cardiomyopathy (32). In the retina of diabetic mice, REDD1-dependent GSK3 activation increases the expression of the proinflammatory factors that act to recruit immune cells into the retina (33). In the current investigation, REDD1-dependent GSK3β activation was required for elevated proinflammatory cytokine expression and subsequent immune cell infiltration in the kidneys of diabetic mice.

Canonical activation of the NF-κB signaling, as seen in disease conditions including sepsis (34) and diabetes (35, 36), involves the phosphorylation of IκB by IKK. Administration of a NEMO-blocking peptide to diabetic mice reduces renal lesions and attenuates albuminuria by preventing IKK holoenzyme assembly and the subsequent NF-κB-mediated inflammatory response (37, 38). We previously reported that both REDD1 and GSK3β are required for activation of IKK in retinal Müller glia exposed to pro-inflammatory stimuli (33, 35). We also found that increased NF-κB activation in the retina requires a REDD1-dependent increase in IKK complex assembly (35). In this study, enhanced phosphorylation of NEMO in renal tissue from diabetic mice was dependent on both REDD1 and GSK3 activity. REDD1 was required for an increase in NEMO binding to IKKβ and GSK3β in response to hyperglycemic conditions. GSK3β was also required for increased IKK assembly in cells exposed to hyperglycemic conditions. Alanine substitution at the GSK3 phosphorylation sites on NEMO (3) revealed that S8 and S31 of NEMO were required for increased GSK3β and IKKβ co-IP in cells exposed to hyperglycemic conditions. Moreover, expression of S8A, S17A, S31A, or S43A variants of NEMO was sufficient to prevent the suppressive effect of hyperglycemic conditions on IκB protein abundance. The observation supports a dominant negative effect of NEMO variants with alanine substitutions at their GSK3 phosphorylation sites, as endogenous NEMO was no longer sufficient to reduce IκB levels in response to hyperglycemic conditions. In addition to enhancing canonical NF-κB signaling *via* GSK3-dependent IKK assembly, it is also important to note that REDD1 may also drive atypical NF-κB activation independently of IKK (39, 40).

While consistent evidence supports that GSK3β is essential for TLR-mediated NF-κB activation, there is also evidence to suggest that GSK3β regulates another proinflammatory signaling mechanism independently of the IKK complex. GSK3β forms an *in vivo* complex with NF-κB1/p105 of the non-canonical NF-κB signaling pathway (41). Under normal physiological conditions, GSK-3β is required to stabilize p105; however, suppression of GSK3β also prevents TNFα-induced processing of p105 to p50 (41). Alternatively, in activated T cells, GSK3β regulates NF-κB activation by directly interacting with RelB to promote its phosphorylation at S552 (42) or S573 (43) and RelB proteolysis. Furthermore, GSK3β also acts as a novel regulator of the antigen receptor-induced CARMA1-BCL10-MALT1 complex, resulting in canonical NF-κB activation (43).

GSK3β likely functions as the dominant isoform in immune regulation in DN. Specifically, GSK3α knockdown is not sufficient to prevent impaired renal function and pathology in diabetic mice (44). By contrast, GSK3α signaling regulates M1 macrophage polarization and promotes atherosclerosis development (45). Importantly, podocyte GSK3α is essential for key cellular processes that augment podocyte health (13). It is worthwhile to note that while pharmacological inhibitors of GSKβ are beneficial, to date, no isoform-selective GSK3 inhibitors have been made commercially available.

A major limitation of the current standards of care for DN is that they predominantly focus on controlling blood glucose levels, and fail to address the specific cellular signaling events that cause renal injury. The studies here provide new insight into the molecular events that contribute to renal inflammation caused by diabetes. Podocytes perform immune-surveillance functions and initiate immune responses that make the glomerular filtration barrier vulnerable to inflammatory disorders like DN (24). The proof-of-concept studies here are consistent with a mechanism of action whereby REDD1 drives renal injury by promoting GSK3β-dependent NF-κB activation in podocytes, thereby enhancing the renal pro-inflammatory immune response to diabetes. Thus, interventions targeting REDD1 or GSK3β in the context of nephropathies linked to metabolic illnesses like diabetes could potentially improve the current standard of care.

## Experimental methods

### Animals

All procedures adhered to the National Institutes of Health Guide for the Care and Use of Laboratory Animals and were approved by the Penn State College of Medicine Institutional Animal Care and Use Committee. Mice were maintained as littermate cages (4 mice/cage) on a 12:12-h reverse light-dark cycle with ad libitum access to food and water. At 6 weeks of age, littermates were randomly divided into treatment groups, and mice were administered either 50 mg/kg streptozotocin (STZ) to induce diabetes or equivalent volumes of sodium citrate (Veh) intraperitoneally for 5 consecutive days. Diabetic phenotype was confirmed by fasting blood glucose concentrations >250 mg/dl. Male mice were used for all studies as female mice were resistant to diabetes induction with the above low-dose STZ protocol and had higher mortality with prolonged study duration at increased STZ dosage. Wild-type (REDD1^+/+^) and REDD1 knockout (REDD1^−/−^) mice on a B6;129 background (46) were made diabetic as described above (n = 8/group, total mice = 32). At 16 weeks of diabetes, mice were euthanized and kidneys were removed, weighed, and processed for examination. GSK3β activity was inhibited in diabetic and non-diabetic C57BL/6J mice (The Jackson Laboratory) by administering daily intraperitoneal injections of either VP3.15 (10 mg/kg, Medchemexpress, Monmouth Junction, NJ) or vehicle (10% DMSO, 0.9% NaCl) during the last 3 weeks of diabetes (n = 4/group, total mice = 16). Protein excretion and urine concentrations of albumin and creatinine were determined from spot urine samples as described (14).

### Cell culture

Conditionally immortalized human podocytes (CIHP-1) were cultured as described previously (14). Human leukemia monocytic THP-1 cells (ATCC TIB-202) were differentiated into macrophages using 50 ng/ml phorbol 12-myristate-13-acetate (PMA) for 48 h. CRISPR/Cas9 genome editing was used to generate a stable CIHP-1 cell line deficient in REDD1 (14). CIHP-1 cells stably expressing a shRNA targeting GSK3β (Fig. S1) were generated as described previously (33). Cells expressing pLKO.1-TRC (Addgene plasmid # 10879) were used as a shRNA control. To model hyperglycemia, cells were exposed to a culture medium containing 30 mM glucose *versus* 5 mM glucose supplemented with 25 mM mannitol as an osmotic control.

### NF-**κ**B luciferase reporter assay

Differentiated CIHP-1 cells were co-transfected with the pRL-CMV Renilla luciferase (Promega) and NF-κB-TATA-luciferase (kindly provided by Dr Edward Harhaj, Penn State College of Medicine) plasmids using Jet PRIME (Polyplus transfection). After 24 h, transfection media was removed, and cells were exposed to hyperglycemic conditions for 48 h. Luciferase activity was measured on a FlexStation3 (Molecular Devices) using a Dual-Luciferase Assay Kit (Promega).

### Immunoprecipitations

Immunoprecipitations were performed on 1000*g* supernatant fractions of cell lysates from differentiated CIHP cells transfected with hemagglutinin (HA)-tagged NEMO (gift from Dr Kun-Liang Guan, Addgene plasmid # 13512) with or without mutations to GSK3 phosphorylation sites using Jet PRIME (Polyplus-transfection). NEMO variants were generated by site-directed mutagenesis using the primers listed in Table S2 and a QuikChange Lightning Kit (Agilent). All plasmids were validated by sequencing analysis. Cells were harvested in lysis buffer [1 mM EDTA, 5 mM EGTA, 10 mM MgCl2, 50 mM β-glycerophosphate, and 0.3% CHAPS] supplemented with 10 mM sodium pyrophosphate, 1 mM benzamidine, 200 mM sodium vanadate, and protease inhibitor mixture (Sigma, 10 μl/ml) and lysed for 30 min at 4 °C. Cell supernatants were collected by centrifuging lysates for 5 min at 1000*g*. HA-tag immunoprecipitation was performed using EZview Red Anti-HA affinity gel (Sigma) overnight at 4 °C. Affinity resins were washed thrice with cold lysis buffer, resuspended in 1× SDS sample buffer, and boiled for 5 min. The immunoprecipitate was subjected to Western blot analysis.

### Protein analysis

Nuclear or total proteins were extracted from cells or renal cortical tissue. Western blot analysis was carried out as described previously (35) with the appropriate antibodies (Table S1). Uncropped Western blot images are included in Fig. S2. IL-1β protein was determined kidney homogenates using the mouse IL-1 beta/IL-1F2 Quantikine ELISA Kit (R&D systems). Nuclear NF-κB activity from renal tissue was quantified using an NF-κB p65 DNA-binding ELISA (TransAM NF-κB p65; Active Motif) (33). Differentiated WT and REDD1 KO CIHP cells (10^5^ cells) were exposed to hyperglycemic conditions for 48 h, and human IL-1β and VEGFA levels in cell culture supernatants were determined using DuoSet ELISA kits (R&D Systems).

### Immunohistology

Renal sections or cells were processed for immunohistochemistry or immunofluorescence (IF) staining as described previously (14). Antibodies are listed in Table S1. Tissue sections were counterstained with Hoechst33342 or hematoxylin to visualize nuclei. Micrographs were captured using either an Amscope T720Q compound microscope or a Leica SP8 confocal laser microscope. Single-channel images in black and white are shown in the Supplemental Information (Fig. S3).

### Transwell migration assay

Migration of THP-1 cells across transwell inserts was measured as described previously (47). The lower chamber of a Transwell was seeded with differentiated CIHP-1 cells, which were then exposed to either an osmotic control or hyperglycemic condition for 48 h. Activated THP-1 cells were transferred into the top chamber of the inserts and allowed to migrate for 24 h. Cells migrating to the bottom surface were stained with 0.1% crystal violet and micrographed using a Nikon Eclipse TS100 inverted microscope. Five fields of view were imaged per sample. The number of migrated cells was quantified using ImageJ software and counts were manually verified.

### Statistical analysis

Data are expressed as mean ± SD. Results from experiments with more than two groups were compared by one-way or two-way ANOVA, with Tukey's test for multiple comparisons used for pairwise analysis. The relationships between urine albumin to creatinine ratio (ACR) and blood glucose levels were tested by Spearman’s correlation analysis. Significance was defined as *p* < 0.05 for all analyses. The sample size for each experiment and exact *p*-values for significantly different groups are listed in Table S3.

## Data availability

All data for this publication are included in the article or are available from the corresponding author upon request.

## Supporting information

This article contains supporting information.

## Conflict of interest

The authors declare that they have no conflicts of interest with the contents of this article.
