# Supplementary material for: REDD1-dependent GSK3β signaling in podocytes promotes canonical NF-κB activation in diabetic nephropathy
Source: J Biol Chem. 2025 Jan 27;301(3):108244. doi: 10.1016/j.jbc.2025.108244 (PMC11904504; doi:10.1016/j.jbc.2025.108244)

Supplemental Information

REDD1-dependent GSK3β signaling in podocytes promotes canonical NF-κB activation in diabetic nephropathy

Sunilkumar et al.

Table of Contents:

1. Table S1: Antibody information
2. Table S2: Mutagenesis primer sequence
3. Table S3: Descriptive statistical analysis
4. Figure S1. GSK3β knockdown validation
5. Figure S2. Uncropped Western Blot Images
6. Figure S3. Immunofluorescence Single channel Images

Table S1. Antibody list

Antibody list					
Assay	Antibody	Source	Dilution	Catalog #	Lot#
Western Blotting	Phospho-GSK3β (Ser 9)	Cell Signaling	1:1000	5558	10
	GSK3β		1:1000	12456	10
	GSK3α/β		1:1000	5676	4
	Phospho-glycogen synthase (Ser 641)		1:1000	3891	2
	Glycogen Synthase		1:500	3886	3
	Phospho-NF-κB p65 (Ser 536)		1:1000	3033	17
	NF-κB p65		1:1000	8242	8
	I-κBα		1:1000	4814	17
	Phospho-IKK α/β (Ser176/180)		1:1000	2697	21
	IKKβ		1:1000	2678	2
	Phospho-NEMO (Ser 376)		1:1000	2689	3
	NEMO		1:1000	2685	3
	CCL2/MCP-1		1:1000	2029	2
	IL-1-beta		1:1000	12242	4
	HA-tag		1:1000	3724	10
	Actin		1:1000	4970	19
	Lamin B	Santa Cruz	1:1000	sc6216	C1307
	GAPDH		1:2000	sc-47724	H1021
	Goat anti-Rabbit IgG h+l-HRP	Bethyl Laboratories	1:10000	A120-101	44
	Goat anti-mouse IgG h+l-HRP		1:10000	A90-116	43
	Rabbit anti-goat IgG h+l-HRP		1:10000	A50-100P	29
IF / IHC	F4/80	Cell Signaling	1:200	30325	3
	Phospho-GSK3β (Ser 9)		1:500	5558	10
	NF-κB p65		1:500	8242	8
	Myeloperoxidase	RA BioSource	1:1000	MM051	D4084
	Donkey anti-Rabbit Alexa Fluor 488	Jackson	1:500	705-546-147	122088
	Donkey anti-Rabbit Alexa Fluor 647	Jackson	1:500	711-605-152	164324

Table S2. Mutagenesis primer sequence

NEMO mutagenesis PCR primers		
Mutation Sites	Forward sequence (5' - 3')	Reverse sequence (5' - 3')
S8A	CCATCTCACACAGTTGGGCCTTCCAGAGGTGCCTAT	ATAGGCACCTCTGGAAGGCCCAACTGTGTGAGATGG
S17A	TGCCGGGCCACCAGCGGGCTGCACCATC	GATGGTGCAGCCCGCTGGTGGCCCGGCA
S31A	CTTCCCCAGAGGAGCCTCTTCGCCCAGTA	TACTGGGCGAAGAGGCTCCTCTGGGGAAG
S43A	GCGCCCTGTTCTGCAGGCAGGTGCAGC	GCTGCACCTGCCTGCAGAACAGGGCGC

Table S3. Descriptive statistics

Figure #	Experimental Groups	Adjusted P Value
Figure 1A	FBG vs. p-GSK3 (n=20) R squared = 0.4816	0.0007
Figure 1C	REDD1+/+ Veh (n=5) vs. REDD1+/+ STZ (n=6)	0.001
Figure 1C	REDD1+/+ STZ (n=6) vs. REDD1-/- STZ (n=6)	0.0345
Figure 1D	REDD1 vs. p-GSK3 (n=12) R squared = 0.707	0.0006
Figure 1E	CIHP WT OC (n=4) vs. CIHP WT HG (n=4)	0.0243
Figure 1E	CIHP WT HG (n=4) vs. CIHP REDD1 KO HG (n=4)	0.0073
Figure 1F	EV OC (n=3) vs. HA-REDD1 OC (n=3)	<0.0001
Figure 1F	EV HG (n=3) vs. HA-REDD1 HG (n=3)	<0.0001
Figure 2A	CIHP shScr OC (n=6) vs. CIHP shScr HG (n=6)	<0.0001
Figure 2A	CIHP shScr HG (n=6) vs. CIHP shGSK3β HG (n=6)	0.0006
Figure 2C	CIHP shScr OC (n=6) vs. CIHP shScr HG (n=6)	<0.0001
Figure 2C	CIHP shScr HG (n=6) vs. CIHP shGSK3β HG (n=6)	<0.0001
Figure 2D	CIHP shScr OC (n=6) vs. CIHP shScr HG (n=6)	<0.0001
Figure 2D	CIHP shScr HG (n=6) vs. CIHP shGSK3β HG (n=6)	0.0003
Figure 2E	CIHP shScr OC (n=4) vs. CIHP shScr HG (n=4)	<0.0001
Figure 2E	CIHP shScr HG (n=4) vs. CIHP shGSK3β HG (n=4)	<0.0001
Figure 2F	CIHP shScr OC (n=4) vs. CIHP shScr HG (n=4)	<0.0001
Figure 2F	CIHP shScr HG (n=4) vs. CIHP shGSK3β HG (n=4)	0.0032
Figure 2H	EV OC (n=6) vs. HA-REDD1 OC (n=6)	<0.0001
Figure 2H	EV HG (n=6) vs. HA-REDD1 HG (n=6)	<0.0001
Figure 3A	REDD1+/+ Veh (n=6) vs. REDD1+/+ STZ (n=6)	0.0008
Figure 3A	REDD1+/+ STZ (n=6) vs. REDD1-/- STZ (n=6)	0.0002
Figure 3B	REDD1+/+ Veh (n=6) vs. REDD1+/+ STZ (n=6)	0.0045
Figure 3B	REDD1+/+ STZ (n=6) vs. REDD1-/- STZ (n=5)	0.0032
Figure 3C	CIHP WT OC (n=6) vs. CIHP WT HG (n=6)	<0.0001
Figure 3C	CIHP WT HG (n=6) vs. CIHP REDD1 KO HG (n=6)	0.0041

Figure #	Experimental Groups	Adjusted P Value
Figure 3E	CIHP shScr OC (n=6) vs. CIHP shScr HG (n=6)	0.0187
Figure 3E	CIHP shScr HG (n=6) vs. CIHP shGSK3β HG (n=6)	0.0075
Figure 4A	CIHP OC DMSO (n=5) vs. CIHP HG DMSO (n=5)	0.0014
Figure 4A	CIHP HG DMSO (n=5) vs. CIHP HG VP3.15 (n=5)	0.0002
Figure 4B	CIHP OC DMSO (n=5) vs. CIHP HG DMSO (n=5)	<0.0001
Figure 4B	CIHP HG DMSO (n=5) vs. CIHP HG VP3.15 (n=5)	<0.0001
Figure 4C	CIHP OC DMSO (n=4) vs. CIHP HG DMSO (n=4)	<0.0001
Figure 4C	CIHP HG DMSO (n=4) vs. CIHP HG VP3.15 (n=4)	<0.0001
Figure 4D	CIHP OC DMSO (n=4) vs. CIHP HG DMSO (n=4)	<0.0001
Figure 4D	CIHP HG DMSO (n=4) vs. CIHP HG VP3.15 (n=4)	0.0021
Figure 4F	FBG vs. p-GS (DMSO; n=8) R squared = 0.7927	0.003
Figure 4F	FBG vs. p-GS (VP3.15; n=7) R squared = 0.8853	0.0016
Figure 4H	Veh DMSO (n=3) vs. STZ DMSO (n=3)	<0.0001
Figure 4H	STZ DMSO (n=3) vs. STZ VP3.15 (n=3)	0.0001
Figure 4I	Veh DMSO (n=4) vs. STZ DMSO (n=4)	<0.0001
Figure 4I	STZ DMSO (n=4) vs. STZ VP3.15 (n=4)	<0.0001
Figure 4J	Veh DMSO (n=4) vs. STZ DMSO (n=4)	<0.0001
Figure 4J	STZ DMSO (n=4) vs. STZ VP3.15 (n=4)	0.0003
Figure 5B	CIHP shScr OC (n=3) vs. CIHP shScr HG (n=3)	<0.0001
Figure 5B	CIHP shScr HG (n=3) vs. CIHP shGSK3β HG (n=3)	<0.0001
Figure 5C	Veh DMSO (n=3) vs. STZ DMSO (n=3)	<0.0001
Figure 5C	STZ DMSO (n=3) vs. STZ VP3.15 (n=3)	<0.0001
Figure 5D	Veh DMSO (n=3) vs. STZ DMSO (n=3)	<0.0001
Figure 5D	STZ DMSO (n=3) vs. STZ VP3.15 (n=3)	<0.0001

A.

shRNA	REFSEQ ID	Oligonucleotide sequence
shScr	pLKO.1-TRC control (RRID:Addgene_10879)	CCGCAGGTATGCACGCGT
shGSK3β #1	NM_002093.3,NM_001146156.1	CCGGCCCAAATGTCAAACTACCAAACTCGAGTTTGGTAGTTTGACATTTGGGTTTTT
shGSK3β #2	NM_002093.3,NM_001146156.1	CCGGCCGATTGCGTTATTTCTTCTACTCGAGTAGAAGAAATAACGCAATCGGTTTTT
shGSK3β #3	NM_002093.3,NM_001146156.1	CCGGCCAATGTTTCGTATATCTGTTCTCGAGAACAGATATACGAAACATTGGTTTTT

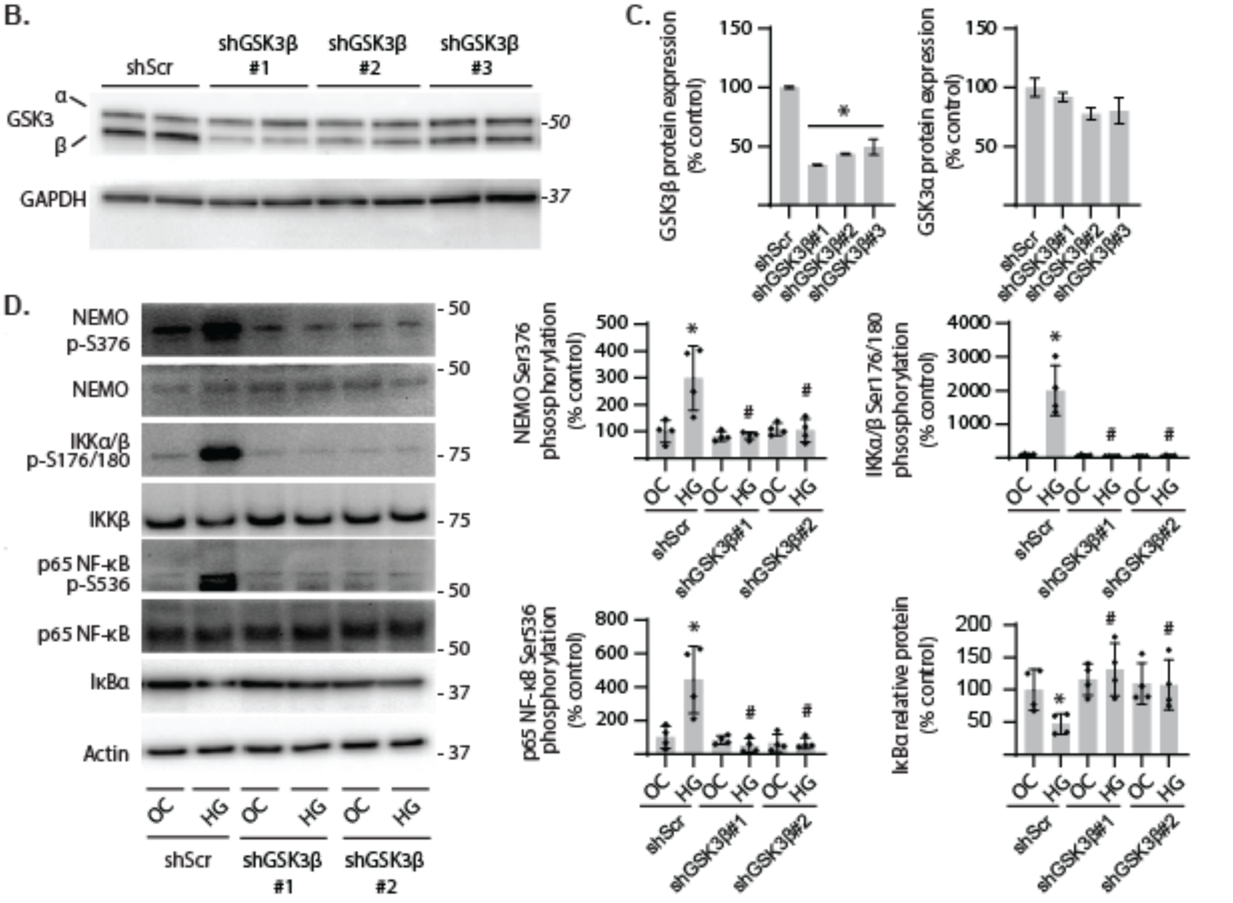

Figure S1. GSK3β knockdown validation in podocyte cultures. *A*, Oligonucleotide sequences and Reference Sequence Identifiers (REFSEQ ID) for shRNAs. *B*, Western blotting was used to evaluate the expression of GSK3 in human podocyte cell lines stably expressing shRNAs targeting GSK3β (shGSK3β) or a scramble shRNA (shScr) control (C). *D,* Cells were exposed to media containing either 30 mM glucose (HG) or an osmotic control (OC; 11.5 mM glucose plus 18.5 mM mannitol) for 48 h. Phosphorylation of NF-κB at Ser536, IKKα/β at Ser176/180, and NEMO at Ser376 was assessed by western blotting. Protein content of NF-κB, IKKβ, NEMO, I-κB, and actin were determined in cell lysates, and relative protein levels were quantified. Molecular mass is indicated to the *right* of the blots. Significance was analyzed by one-way ANOVA with pairwise comparisons made using Tukey's test for multiple comparisons. *, $p < 0.05$ versus OC; # $p < 0.05$ versus shScr.

Figure S2. Uncropped Western Blot Images

Figure 1C

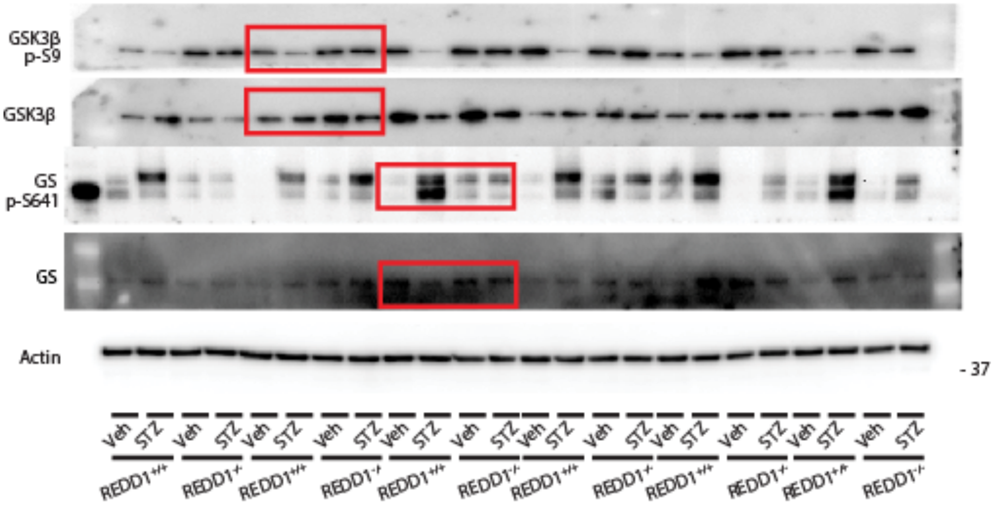

Figure 1E

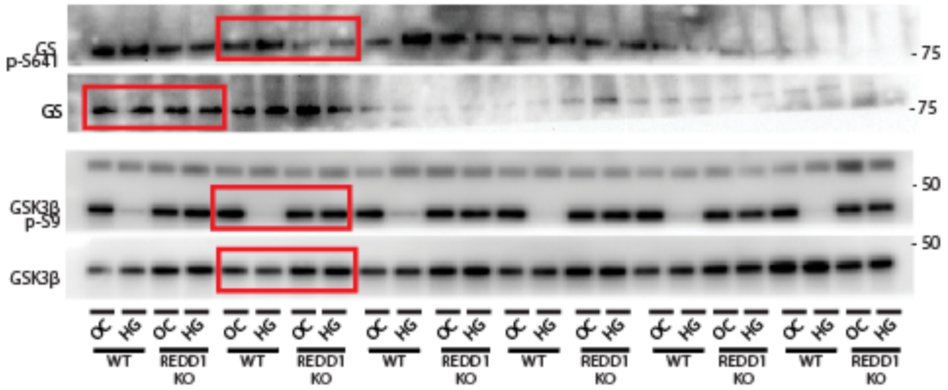

Figure 1F

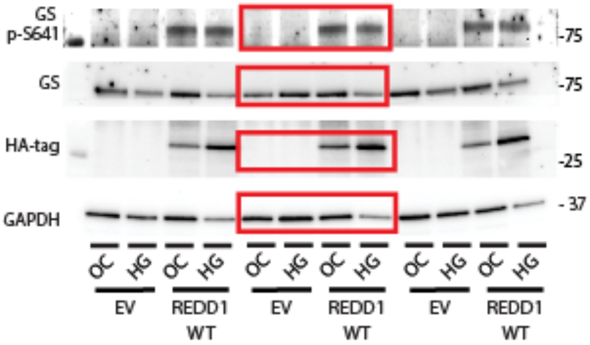

Figure 2A

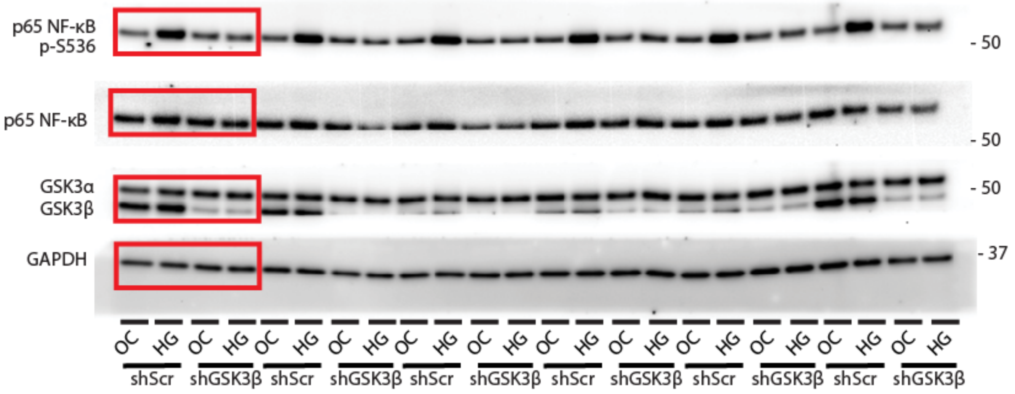

Figure 2D

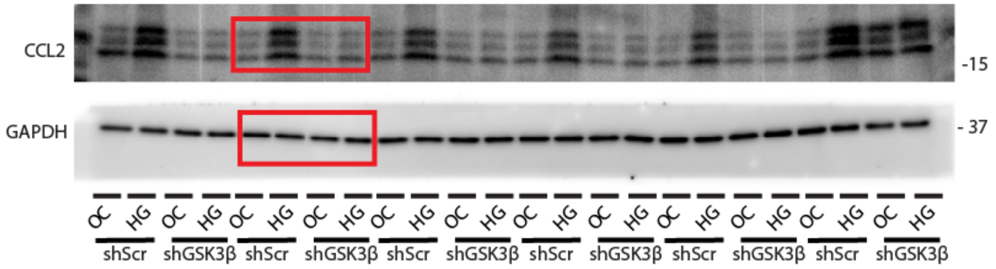

Figure 2G

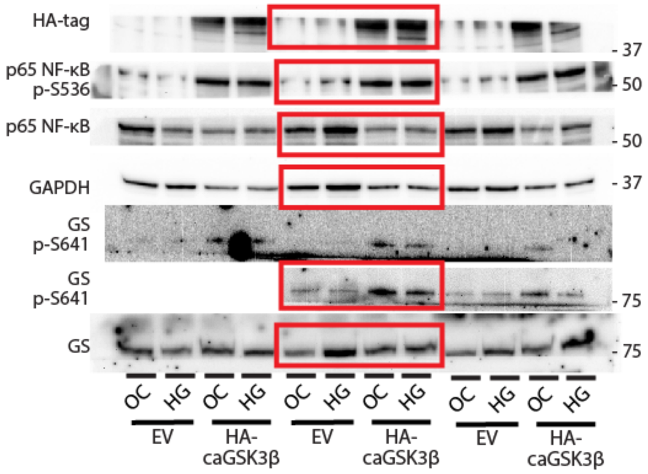

Figure 3A

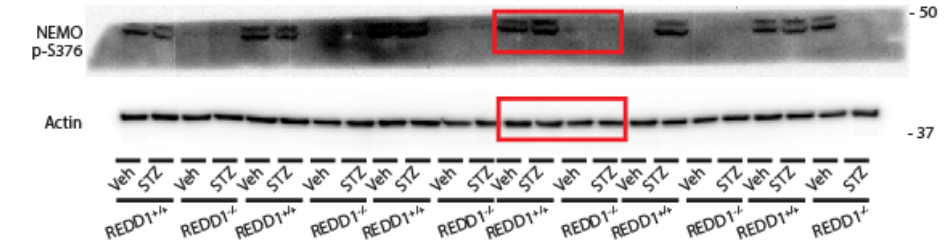

Figure 3B

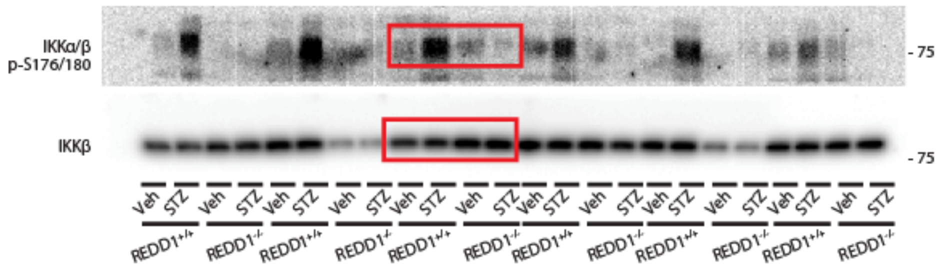

Figure 3C

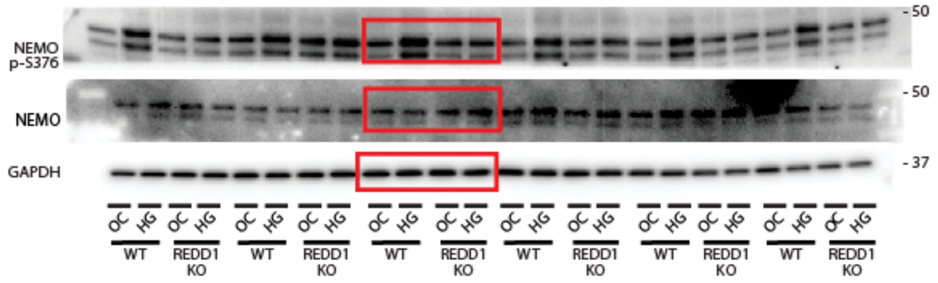

Figure 3D

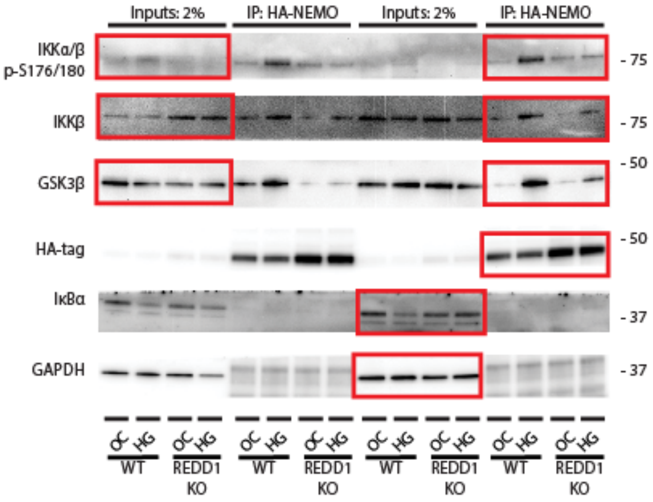

Figure 3E

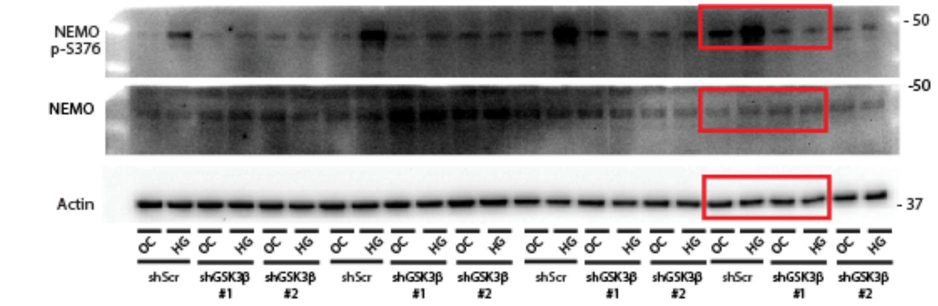

Figure 3F

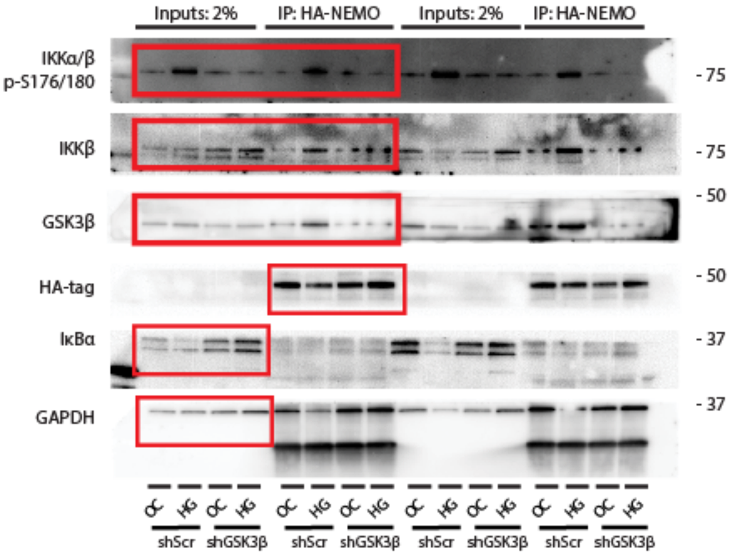

Figure 3G

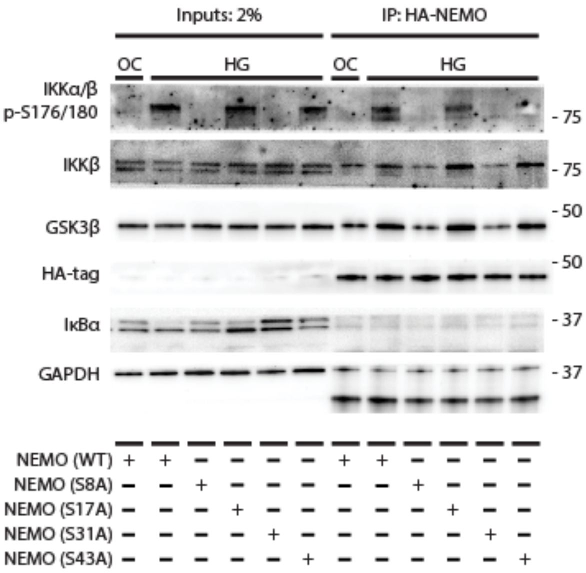

Figure 4A

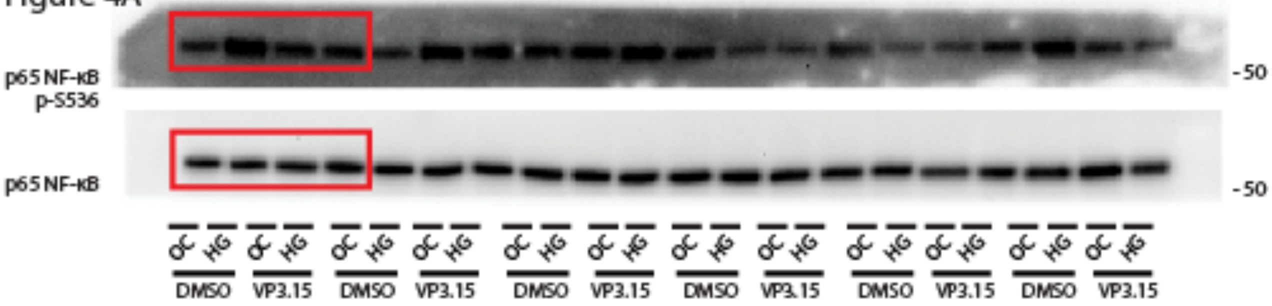

Figure 4B

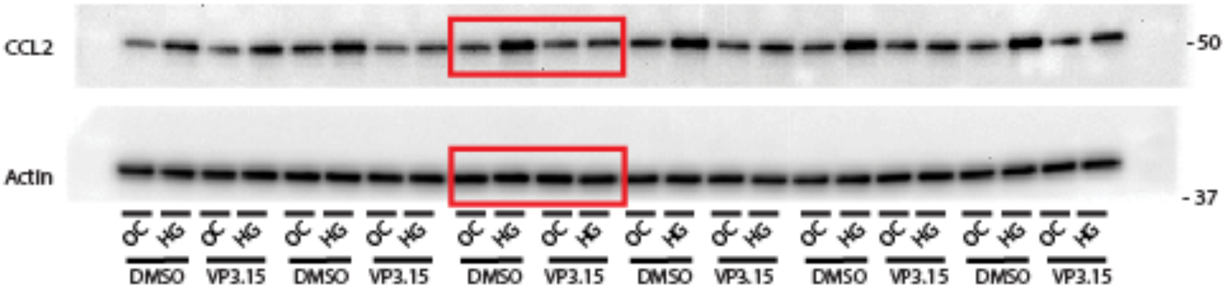

Figure 4G

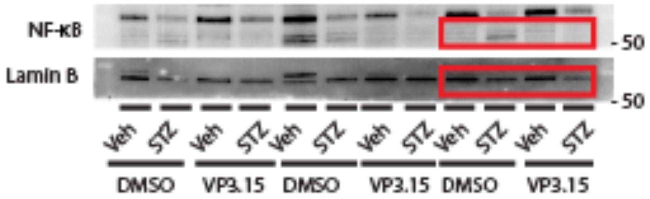

Figure 4G

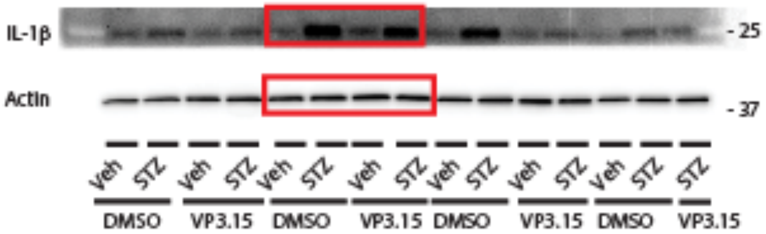

Figure 4J

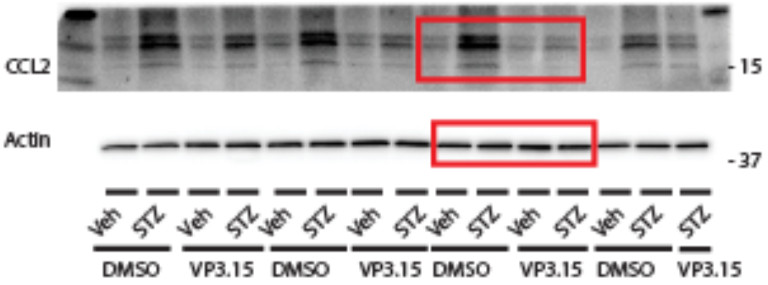

Figure S1D

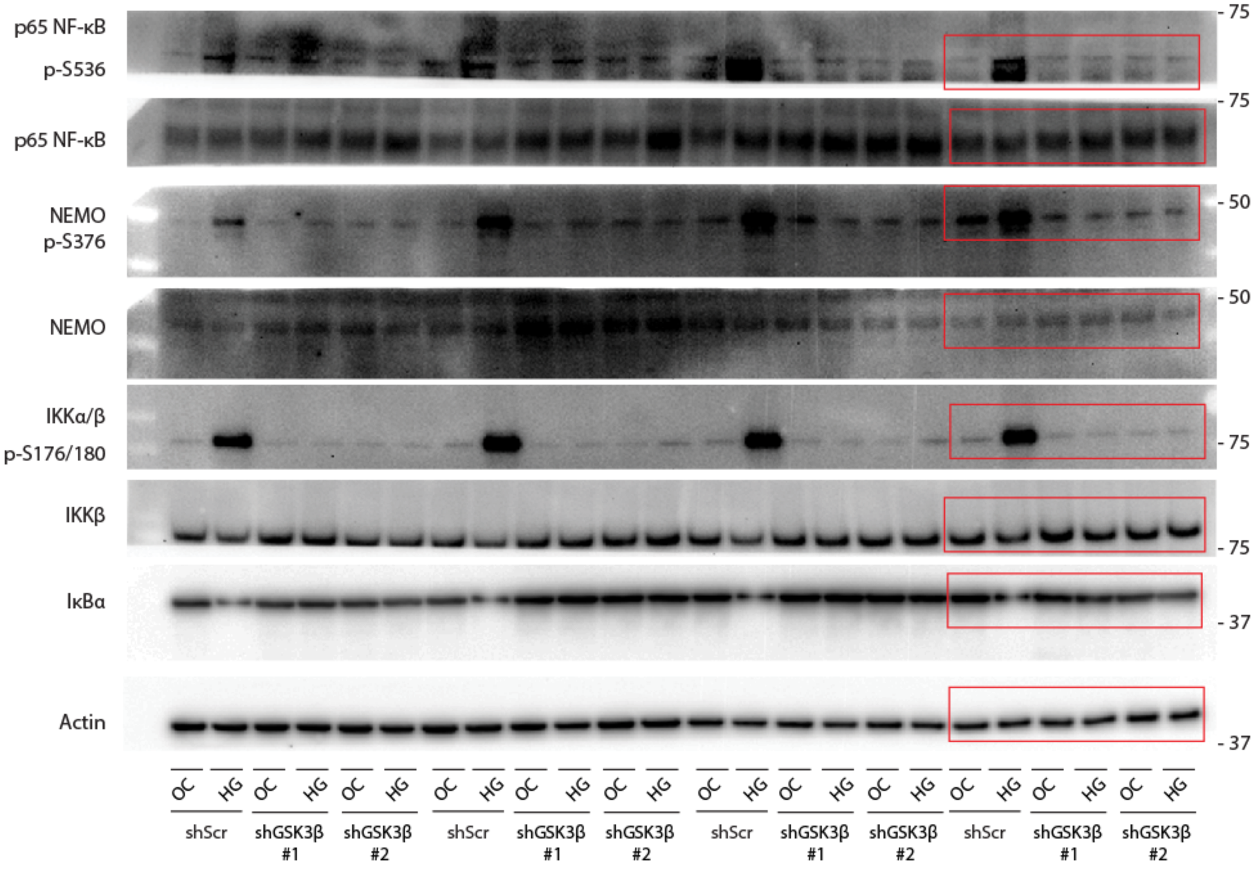
p65 NF-κB
p-S536
p65 NF-κB
NEMO
p-S376
NEMO
IKKα/β
p-S176/180
IKKβ
IκBα
Actin
- 75
- 75
- 50
- 50
- 75
- 75
- 37
- 37
OC
HG
shScr
shGSK3β #1
shGSK3β #2

Figure S3. Immunofluorescence Single Channel Images

Figure 5A

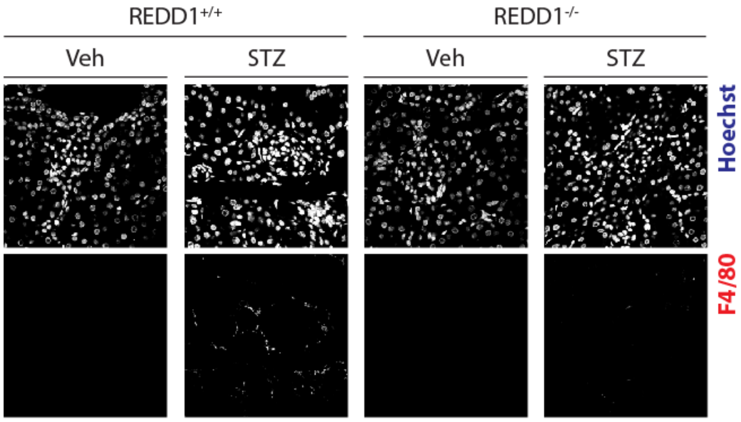

Figure 5D

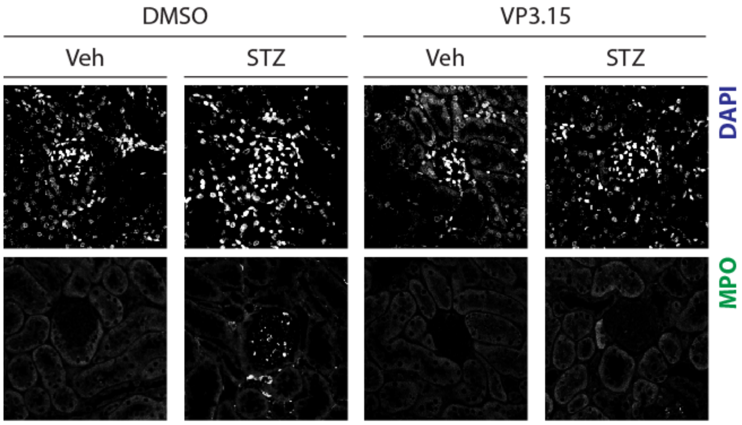

Supplement: Supporting information [file mmc1.pdf]
